# Gut microbiota links to histological damage in chronic HBV infection patients and aggravates fibrosis via fecal microbiota transplantation in mice

**DOI:** 10.1128/spectrum.00764-25

**Published:** 2025-07-11

**Authors:** Fengjiao Wang, Yechen Wu, Jiali NI, Qianhan Xie, Jian Shen, Hui Chen, Chengyu Ma, Yuanyuan Yao, Jinzhi Wang, Lvwan Xu, Qiangqiang Xiang, Yuxi Zhao, Yanfei Chen, Lanjuan Li

**Affiliations:** 1 Shandong First Medical University 518873, Jinan, China; 2 Jinan Microecological Biomedicine Shandong Laboratory 661980 , Jinan, China; 3 State Key Laboratory for Diagnosis and Treatment of Infectious Diseases, National Clinical Research Center for Infectious Diseases, National Medical Center for Infectious Diseases, Collaborative Innovation Center for Diagnosis and Treatment of Infectious Diseases, The First Affiliated Hospital, Zhejiang University School of Medicine 26441 , Hangzhou, China; 4 Cancer Center, Department of Gastroenterology, Zhejiang Provincial People's Hospital (Affiliated People's Hospital), Hangzhou Medical College 117839, Hangzhou, Zhejiang, China; 5 The First Affiliated Hospital of Henan University of Science and Technology 159366, Luoyang, China; Children's National Hospital, George Washington University, Washington, DC, USA

**Keywords:** HBV, liver biopsy, fecal microbiome transplantation, gut microbiota, liver fibrosis

## Abstract

Gut microbiota dysbiosis has been observed in HBV-related cirrhosis, but its role in early-stage disease and its correlation with liver pathology remain unclear. Moreover, whether dysbiosis is a cause or consequence of liver cirrhosis is still debated. We recruited 20 treatment-naïve patients with chronic HBV infection, assessing liver injury via biopsy. Fecal metagenomic sequencing was used to analyze the correlation between gut microbiota and liver histology. To explore the causality, fecal samples from an HBV-related cirrhosis patient were transplanted into mice with CCl₄-induced liver fibrosis. Patients with significant histological damage exhibited reduced alpha diversity and greater microbial homogeneity. Species such as *Eubacterium_sp_CAG_180*, *Gemmiger_formicilis*, and *Oscillibacter_sp_ER4* had decreased abundance, while *Parabacteroides_distasonis*, *Bacteroides_dorei*, and *Bacteroides_finegoldii* were enriched. Mice receiving fecal transplants from the cirrhotic patient showed aggravated liver fibrosis, with increased collagen deposition; elevated ALT, AST, and ALP levels; and heightened hepatic inflammatory gene expression. Additionally, abnormal bile acid profiles with elevated unconjugated bile acids (e.g., GCA and CA) were observed. Gut microbiota dysbiosis is closely associated with liver histological damage in chronic HBV infection and may drive fibrosis progression via microbial-bile acid interactions. These findings suggest potential for gut microbiota-based assessment and treatment strategies in chronic hepatitis B.

**IMPORTANCE:**

This study elucidates a significant association between gut microbiota dysbiosis and liver histological damage in patients with chronic hepatitis B (HBV), potentially exacerbating fibrosis progression through bile acid interactions. By analyzing patient gut microbiota and conducting fecal transplant experiments in mice, researchers have identified that gut microbiota dysbiosis contributes to hepatic fibrosis during chronic HBV infection. These findings underscore the importance of the gut-liver axis in HBV disease progression, indicating that monitoring or modulating gut bacteria may facilitate early diagnosis or therapeutic interventions. This research bridges the gap in understanding whether microbial alterations drive disease progression or result from it, providing a foundation for developing therapies targeting the microbiome to mitigate liver damage in chronic HBV infections.

## INTRODUCTION

Hepatitis B virus (HBV) infection remains a major global public health challenge, with an estimated 296 million people living with chronic hepatitis B (CHB) worldwide, as of 2019 (1). Despite the availability of effective vaccines and antiviral therapies, HBV continues to cause significant liver-related morbidity and mortality, leading to over 800,000 deaths annually due to complications such as cirrhosis, liver failure, and hepatocellular carcinoma (HCC)(2).

The clinical course of HBV infection varies widely, ranging from inactive carrier states to progressive liver disease, including cirrhosis and HCC. The mechanisms driving CHB progression remain unclear and are influenced by factors such as genetic predisposition, host immune response, HBV genotype, viral load, and age at infection (3). Early identification of these mechanisms is critical for timely interventions to prevent severe liver complications (4, 5).

Emerging evidence suggests that the gut microbiota plays a key role in CHB progression by modulating immune responses and liver inflammation (6). HBV infection alters gut microbiota composition in mice, disrupting microbial community development and shifting the *Firmicutes*/*Bacteroidetes* ratio (7). In persistent HBV infection models, *Akkermansia* levels are significantly reduced but recovered following entecavir treatment, showing a negative correlation with HBV DNA levels in the serum and liver (8). Additionally, while adult mice can rapidly clear HBV, young or antibiotic-treated mice cannot, indicating that gut microbiota maturation is crucial for effective immune responses and HBV clearance (9).

Gut dysbiosis, characterized by imbalances in microbial composition, has been observed in CHB patients, especially those with advanced liver disease. Studies have profiled the gut microbiota across different stages of HBV-related liver disease—including resolved infection, CHB, cirrhosis, and HCC—highlighting dynamic microbial shifts during disease progression (9, 10). Notably, alterations in gut microbiota composition are detectable even in early cirrhosis (10). Wang et al. identified distinct microbial signatures in HBV-ACLF patients, with *Lactobacillus casei paracasei* enriched in progressive cases, while *Alistipes senegalensis*, *Faecalibacterium prausnitzii*, and *Parabacteroides merdae* were dominant in regressive cases (11).

However, most studies focus on advanced CHB stages, where gut microbiota can be influenced by factors such as medications, co-infections, and antiviral therapies. Limited research has explored the correlation between gut microbiota and liver pathology in early-stage CHB. Although liver biopsy is the gold-standard diagnostic measure for assessing liver injury, it is invasive, limiting its widespread use. Previous studies suggest a potential link between gut microbiota and liver inflammation, but no research has directly analyzed the association between gut microbiota and liver histopathology, the definitive marker for liver inflammation and fibrosis. Establishing this correlation could pave the way for developing gut microbiota-based biomarkers for CHB progression.

To address this gap, our study investigated the relationship between gut microbiota composition and liver histological changes—including inflammation and fibrosis—confirmed by liver biopsy in CHB patients. Additionally, we validated the role of gut microbiota in fibrosis progression using a CCl₄-induced liver fibrosis mouse model with fecal microbiota transplantation (FMT) from HBV-related liver fibrosis patients.

## MATERIALS AND METHODS

### Patients

**Fig 1:**
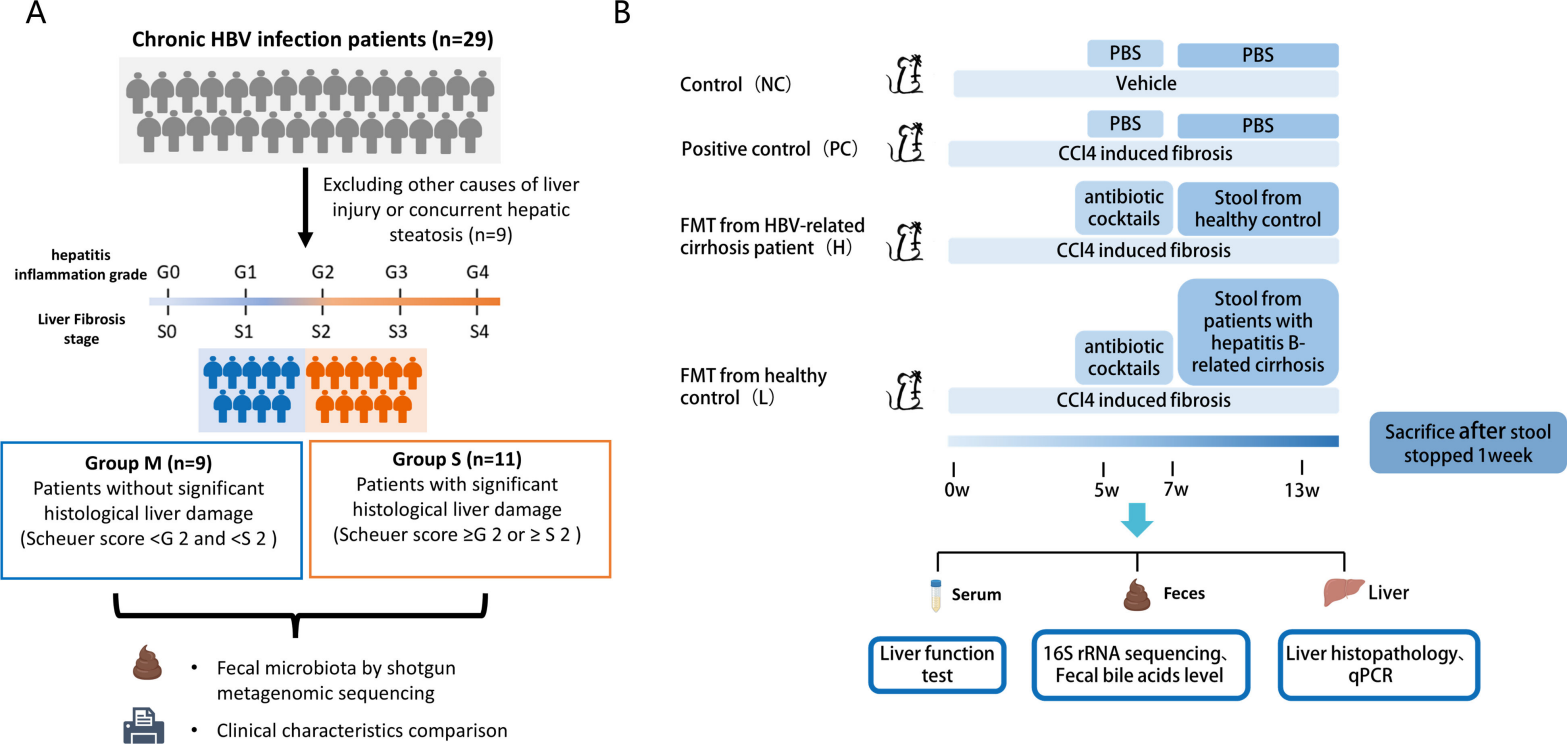
The experiment design scheme. (**A**) Grouping clinical cohort: Group M (minimal liver injury, *n* = 9, defined as G0–1 and S0–1) and Group S (significant liver injury, *n* = 11, defined as G > 1 and/or S > 1) according to Scheuer score. (**B**) Animal experiment: FMT treatment experiment. Group NC: negative controls, mice without CCl4 and fecal microbiota transplantation. Group L: fecal microbiota transplantation from HBV-related cirrhosis patients; Group H: FMT from age- and gender-matched healthy controls as the cirrhotic patients; PC: positive controls, only CCl4 was applied to induce fibrosis, and FMT was not performed. FMT: fecal microbiota transplantation.

This prospective study recruited treatment-naïve chronic HBV patients who underwent liver biopsy at the First Affiliated Hospital of Zhejiang University between January 2021 and December 2021. Inclusion criteria were HBV-infected patients with liver enzyme levels less than onefold the upper limit of normal (ULN) and without liver fibrosis. Exclusion criteria included the following: (i) co-infection with other viruses, including human immunodeficiency virus (HIV), hepatitis C virus (HCV), hepatitis D virus (HDV), Epstein-Barr Virus (EBV), or cytomegalovirus (CMV); (ii) prior antiviral treatment; (3) significant alcohol consumption (≥40 g/day for males, ≥20 g/day for females); (4) cirrhosis or HCC; (5) other chronic liver diseases such as primary biliary cholangitis, primary sclerosing cholangitis, autoimmune hepatitis, or Wilson’s disease (Fig. 1A).

Clinical data, including age, gender, body mass index (BMI), alanine aminotransferase (ALT), aspartate aminotransferase (AST), and HBV DNA levels, were extracted from electronic medical records. The ULN for both ALT and AST was set at 40 U/L for males and females. The study was approved by the Ethics Committee of the First Affiliated Hospital, School of Medicine, Zhejiang University (reference number: IIT 20210312B-R1). Subjects were enrolled in the study based on the ethical guidelines of the 1975 Declaration of Helsinki for the participation of human subjects.

### Liver biopsy

Liver tissue was obtained via percutaneous biopsy using a 16G needle, immediately fixed in 10% formalin, paraffin-embedded, sectioned, and stained with hematoxylin-eosin (H&E) and Masson’s trichrome for histopathological analysis. Two experienced pathologists, blinded to clinical and biochemical data, evaluated the biopsies using the modified Scheuer scoring system (12), including hepatitis inflammation grade (G) and liver fibrosis stage (S): G0–1 (minimal or no liver inflammation); S0–1 (minimal or no liver fibrosis); G0–1 and S0–1 (minimal or no inflammation and fibrosis, considered normal liver histology); G2–4 (significant liver inflammation); S2–4 (significant liver fibrosis); G2–4 or S2–4 (significant liver disease, abnormal histology). Significant histological damage was defined as liver inflammation grade ≥G2 and/or fibrosis stage ≥S2.

### Animal research

Twenty-four male BALB/c mice (6–8 weeks old) were housed under specific pathogen-free (SPF) conditions with a 12-hour light/dark cycle at 22–25°C. Hepatic fibrosis was induced via intraperitoneal injection of 2.5 µL/g of 20% CCl₄, administered twice weekly. All procedures were approved by the Animal Care and Use Committee of the First Affiliated Hospital, School of Medicine, Zhejiang University (reference number: 2021-1194). After 5 weeks of fibrosis induction, mice were randomized into three groups: 1. positive control; 2. group L: FMT from HBV-related cirrhosis patients; 3. group H: FMT from healthy controls with matched age and gender as the cirrhotic patients. Before FMT, mice were treated with an antibiotic cocktail (ampicillin, vancomycin, neomycin sulfate, and metronidazole) via oral gavage for 1 week to create a pseudo-germ-free environment. Following a 1-week washout period, biweekly FMT was performed for 6 weeks using fresh stool samples from treatment-naïve HBV-cirrhosis patients and age- and gender-matched healthy controls. Fecal samples were homogenized in PBS (1:10 ratio), filtered to eliminate large particles, and prepared as a fecal suspension for gavage. Mice were sacrificed 1 week after the final FMT dose for sample collection (Fig. 1B).

### Metagenomic and 16S rRNA sequencing and analysis

DNA was extracted from stool samples using the PowerSoil Pro Kit (Qiagen) per the manufacturer’s instructions and stored at −20°C. Shotgun metagenomic sequencing was conducted for human fecal samples as described in the previous study (13). Shotgun metagenome sequencing was performed using the Illumina HiSeq 6000 sequencing platform (Beijing Novogene Technology Co., Ltd.). DNA fragments approximately 300 bp in length were prepared from the library, and these reads were trimmed using Sickle software, followed by the removal of the host DNA fragments. All metagenomic data analyses were performed using NovoMagic (https://magic.novogene.com). 16S rRNA sequencing was conducted for murine fecal samples, targeting the V3–V4 region using Illumina MiSeq as described in the previous study (13). Bioinformatic analysis of the 16S rRNA sequencing was carried out using the Majorbio Cloud platform (https://cloud.majorbio.com). Statistical analyses included alpha diversity, beta diversity, principal coordinate analysis (PCoA), and taxonomic differences. The raw sequence data reported in this paper have been deposited in the Genome Sequence Archive (Genomics, Proteomics & Bioinformatics 2021) in the National Genomics Data Center (Nucleic Acids Res 2022), China National Center for Bioinformation/Beijing Institute of Genomics, Chinese Academy of Sciences (GSA: CRA023643 and CRA023641) that are publicly accessible at https://ngdc.cncb.ac.cn/gsa.

### Histology and inflammation analysis of murine tissues after FMT

Murine blood, liver tissue, and feces were collected and stored at −80°C. Liver tissue was used for histological evaluation and cytokine analysis. Total RNA was extracted for reverse transcription PCR (RT-PCR) to quantify the mRNA levels of inflammatory cytokines (IL-1β, IL-6, IL-18, and TNF-α). Primer sequences are listed in Table 1. For bile acid profiling, ultra-performance liquid chromatography-tandem mass spectrometry (UPLC-MS/MS) (AB Sciex/Waters, Shanghai, China) was used to quantify 53 bile acid species (14).

**TABLE 1:** Primer pairs for real-time RT-PCR^*a*^

Genes	Forward 5’→3’	Reverse 5’→3’
β-actin	AGTGTGACGTTGACATCCGT	GCAGCTCAGTAACAGTCCG
IL-1β	GAAATGCCACCTTTTGACAGTG	TGGATGCTCTCATCAGGACAG
IL-6	TCTATACCACTTCACAAGTCGGA	GAATTGCCATTGCACAACTCTTT
IL-18	GCCATGTCAGAAGACTCTTGCGTC	GTACAGTGAAGTCGGCCAAAGTTGTC
TNF-α	AGGGTCTGGGCCATAGAACT	CCACCACGCTCTTCTGTCTAC

^*a*^ TNF-α, tumor necrosis factor-α; IL-1β, interleukin-1β; IL-6, interleukin-6; IL-18, interleukin-18.

### Statistical analysis

Statistical analyses were performed using GraphPad Prism (version 10.0.0), and the results are reported as mean ± SEM. Student’s *t*-test was employed to analyze differences between two groups with normal distribution; otherwise, the Mann-Whitney U test was utilized. For analysis among multiple groups, analysis of variance (ANOVA) was conducted to examine groups with equal standard deviations (SDs); otherwise, Brown-Forsythe and Welch ANOVA were performed. The correlation was calculated using Spearman’s rank correlation coefficient. *P* values < 0.05 were considered statistically significant.

## RESULTS

### Clinical characteristics and liver histology of study patients

**Fig 2:**
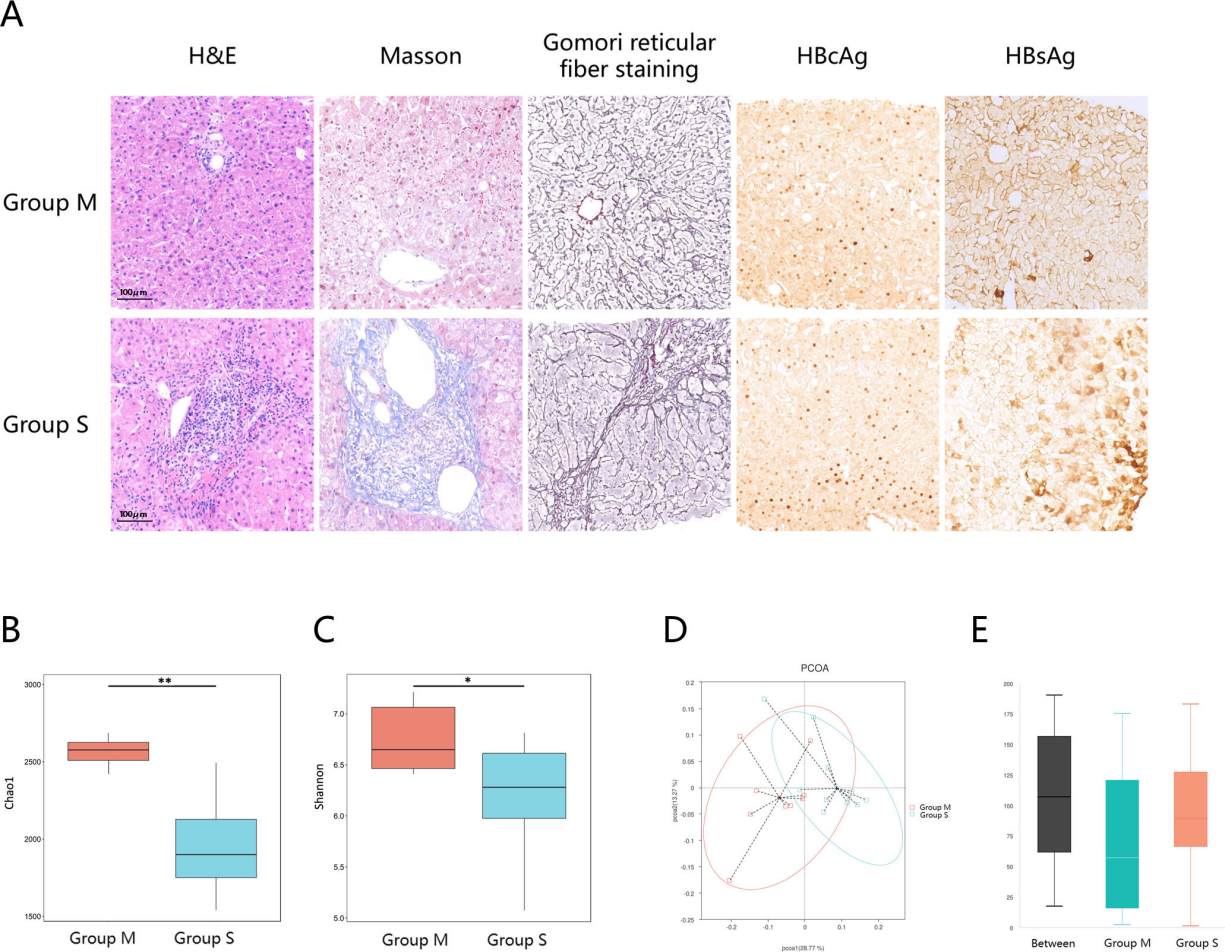
Pathological staining findings of biopsy specimens and metagenomic analysis of fecal microbiota diversity in CHB patients: Group M and Group S. (**A**) Representative images of the inflammation and fibrosis were scored using H&E staining, Masson staining, and Gomori reticular fiber staining. Immunohistochemistry of hepatitis B surface antigen (HBsAg) and hepatitis B core antigen (HBcAg) in the liver with chronic hepatitis (scale bar, 100 µm). Alpha diversity between groups M and S: the Shannon index (B) and Chao1 index (C). (**D**) PCoA of gut microbiota. (**E**) ANOSIM analysis: R = 0.265, *P* = 0.003. Group M: patients with minimal liver injury; Group S: patients with significant liver injury. *P < 0.05; **P < 0.01.

A total of 20 patients were enrolled in the study and divided into two groups based on liver biopsy histological findings: Group M (minimal liver injury, *n* = 9, defined as G0–1 and S0–1), Group S (significant liver injury, *n* = 11, defined as G > 1 and/or S > 1). The pathological findings from the liver biopsy are presented in Fig. 2A. Table 2 summarizes the clinical characteristics of both groups. Patients with significant histological damage (group S) exhibited significantly higher levels of ALT and AST compared to those with minimal liver injury (*P* < 0.05 for both). Interestingly, patients with minimal liver injury (group M) were slightly older than those with significant liver injury, although this difference did not reach statistical significance (*P* = 0.071).

**TABLE 2:** Clinical characteristics of patients in study^*a*^^,*d*^

	Group M (minimal liver injury, *n* = 9)	Group S (significant liver injury, *n* = 11)	*P*-value
Age, years	44.4 ± 9.4	36.8 ± 7.9	0.071
Male, n (%)	7 (77.8%)	7 (63.6%)	0.490
BMI, kg/m^2^	23.1 ± 2.8	21.4 ± 3.3	0.246
HBV-DNA (log^10^ copies /mL）	4 (3, 8)	6 (2.5, 7.75)	0.975
HBeAg-positive	4 (44.4%）	8 (72.7%）	0.768
Histological scoring of the Scheuer system			
Grading, G	1 (1，1）	2 (2，2）	6.334E-07^*b*^
Staging, S	1 (1, 1)	2 (1, 2)	0.001^*b*^
ALT (U/L)	24.4 ± 11.1	41 ± 19.5	0.015^*c*^
AST (U/L)	21.8 ± 9	34.4 ± 15.9	0.02^*c*^
WBC (10^9^ /L)	5.1 ± 0.8	5.7 ± 1.8	0.238
Hgb (g/L)	142.4 ± 13.2	140.2 ± 24.9	0.904
PLT (10^9^ /L)	116.9 ± 48.3	205.5 ± 51.6	0.117

^*a*^ The quantitative data are shown as median data and inter-quartile range data in brackets. The occurrence data are shown as no. (%). Values indicate no. of positive results/total no. of patients with available assay results. Kruskal-Wallis test or Fisher's exact test was used for comparison between mild and severe groups, respectively, when applicable, with p < 0.05 indicating significance.

^*b*^ *P* < 0.01.

^*c*^ *P* < 0.05.

^*d*^ Grading, G: the grade of hepatitis inflammation evaluated by the Scheuer system; Staging, S: the stage of liver fibrosis evaluated by the Scheuer system; BMI, body mass index; HBeAg, hepatitis B e antigen; ALT, alanine transaminase; AST, aspartate transaminase; WBC, white blood cell; Hgb, hemoglobin; PLT, platelet.

### Correlation between the gut microbiota and the grades of liver histological damage

Significant differences in gut microbial alpha diversity were observed between patients with and without significant liver histological damage. Patients with minimal liver injury (group M) demonstrated greater microbial species richness and diversity, as evidenced by a significantly higher Chao1 index (2,485 ± 257 vs 1,897 ± 291, *P* < 0.001) (Fig. 2B) and Shannon index (6.75 ± 0.32 vs 6.24 ± 0.52, *P* = 0.015) (Fig. 2C) than patients in group S.

Beta diversity analysis, visualized through principal coordinate analysis (PCoA) based on UniFrac distances at the species level, revealed partial overlap between the groups but also a clear trend of separation, indicating shifts in the microbial community composition (Fig. 2D). Further analysis using Analysis of Similarities (ANOSIM) showed significantly lower intragroup differences among patients with significant histological damage (R = 0.265, *P* = 0.003), suggesting a more homogeneous microbial community structure in these patients (Fig. 2E).

**Fig 3:**
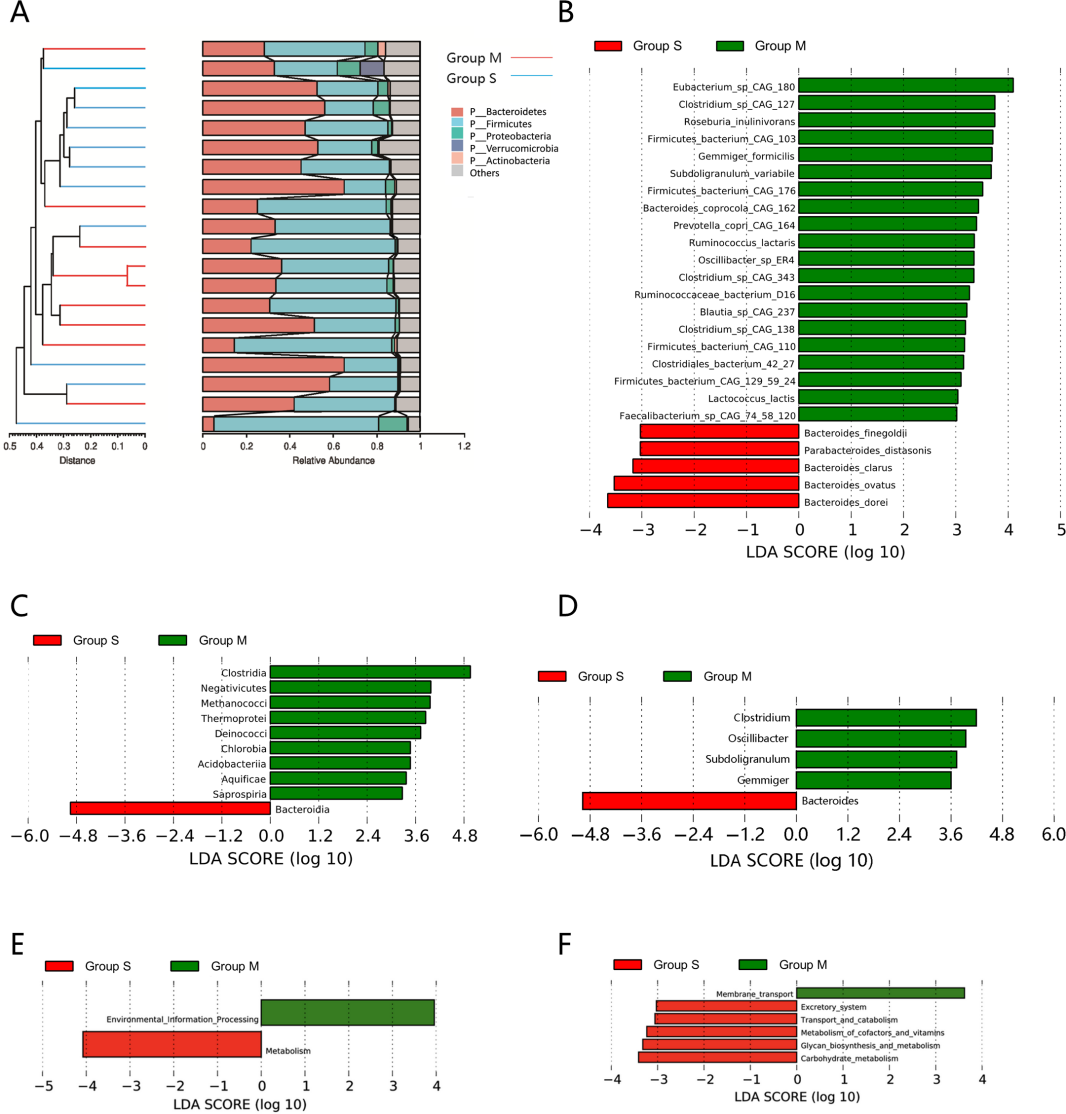
(**A**) Hierarchical cluster tree diagram of the microbial community at the phylum level. Linear discriminant analysis (LDA) coupled with effect size measurements identifies significant abundance. LDA scores > 2 between the two groups are listed at the class level (**C**), genus level (D), and species level (B). The KEGG database predicts the function of the gut microbiota, showing the outcomes of the KEGG pathway at level 1 (**E**) and level 2 (**F**). Group M: patients with minimal liver injury; Group S: patients with significant liver injury. *P < 0.05; **P < 0.01.

At the phylum level, Group M had a higher relative abundance of *Firmicutes*, while Group S exhibited an increased abundance of *Bacteroidetes* (Fig. 3A). At the class level, the predominant contributors to these differences were *Bacteroidia* (from *Bacteroidetes*) and *Clostridia* (from *Firmicutes*), with other differentially abundant classes in Group M, including *Negativicutes* (Fig. 3C). At the genus level, Group M showed higher abundances of *Clostridium*, *Oscillospira*, *Subdoligranulum,* and *Gemmiger*, while Group S was enriched in *Bacteroides* (Fig. 3D).

Further species-level analysis revealed distinct microbial signatures between the two groups (Fig. 3B). Patients in Group M had higher abundances of *Eubacterium_sp_CAG_180*, *Gemmiger_formicilis*, *Oscillibacter_sp_ER4*, *Prevotella_copri_CAG_164*, *Clostridiales_bacterium_42_27*, *Roseburia_inulinivorans*, *Ruminococcus_lactaris*, *Subdoligranulum_variabile*, *Blautia_sp_CAG_237*, *Faecalibacterium_sp_CAG_74_58_120*, and *Lactococcus_lactis*. In contrast, patients in Group S showed enrichment of *Parabacteroides distasonis*, *Bacteroides dorei*, *Bacteroides finegoldii*, *Bacteroides ovatus*, and *Bacteroides clarus*.

Functional profiling of the gut microbiome using KEGG pathway analysis revealed distinct differences between groups. Group M was enriched in genes related to Environmental Information Processing, while Group S showed higher abundance of genes involved in Metabolism. At KEGG level 2, Group S exhibited enrichment in pathways related to Carbohydrate Metabolism, Glycan Biosynthesis and Metabolism, Metabolism of Cofactors and Vitamins, Transport and Catabolism, and the Excretory System. Conversely, Group M had a greater abundance of genes associated with Membrane Transport (Fig. 3E and F).

### FMT from HBV-related cirrhosis patients aggravates hepatic dysfunction and fibrosis in mice

**Fig 4:**
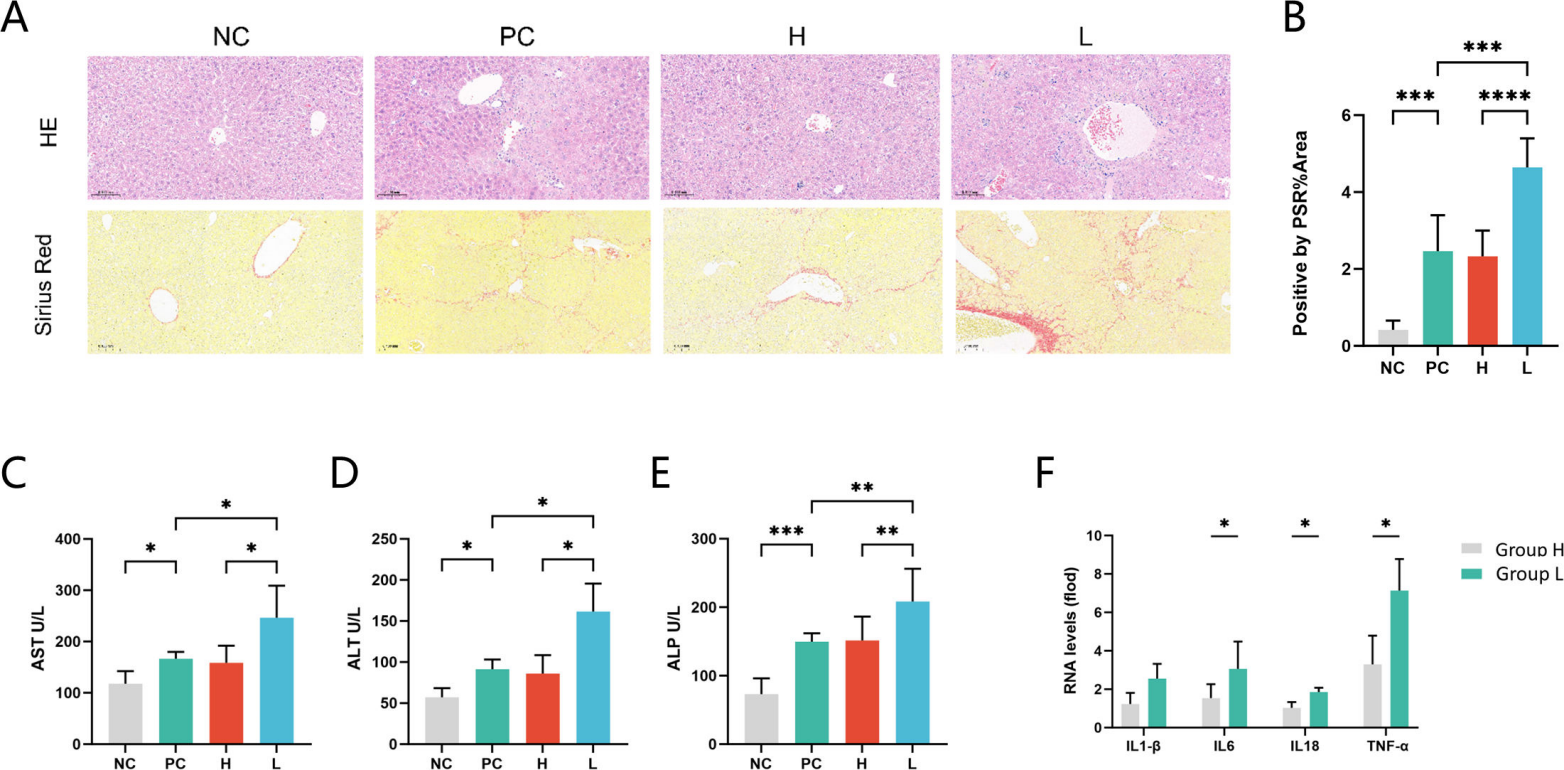
Effects of HBV-related cirrhosis stool on liver fibrosis, liver function, and inflammation levels in the mouse. (**A**) H&E, Sirius red staining of liver sections (scale bar, 100 µm). (**B**) The positive region of Sirius red staining was quantified using ImageJ. Liver function tests of serum: (**C**) AST, (**D**) ALT, and (**E**) ALP. (**F**) Relative expression of cytokines in liver tissues: IL-1β, IL-6, IL-18, and TNF-α). Group NC: negative controls, mice without CCl4, and fecal microbiota transplantation. Group L: fecal microbiota transplantation from HBV-related cirrhosis patients; Group H: FMT from age- and gender-matched healthy controls as the cirrhotic patients; Group PC: positive controls, only CCl4 was applied to induce fibrosis, and FMT was not performed. * *P* < 0.05, **P < 0.01, and ***P < 0.001. Note: Faint gridlines in **Fig. 4A **result from a transient optical calibration anomaly in the digital slide scanner during image acquisition.

FMT from HBV-related cirrhosis patients significantly worsened hepatic dysfunction and inflammation in mice. Histological analysis showed severe inflammatory cell infiltration in mice receiving FMT from a cirrhosis patient (Group L), along with increased collagen deposition and fibrosis, as demonstrated by Sirius Red staining (Fig. 4A and B). This was also evidenced by elevated serum levels of ALT, AST, and ALP compared to controls (Fig. 4C–E). Quantitative PCR revealed that FMT from HBV-cirrhosis patients upregulated the hepatic mRNA expression of inflammatory cytokines, including IL-6, TNF-α, and IL-18 (Fig. 4F). These findings suggest that FMT from the HBV-related cirrhosis patients exacerbates liver dysfunction, inflammation, and fibrosis compared to FMT from the healthy controls.

### Gut microbiota composition in mice after FMT from HBV-related cirrhosis patients

**Fig 5:**
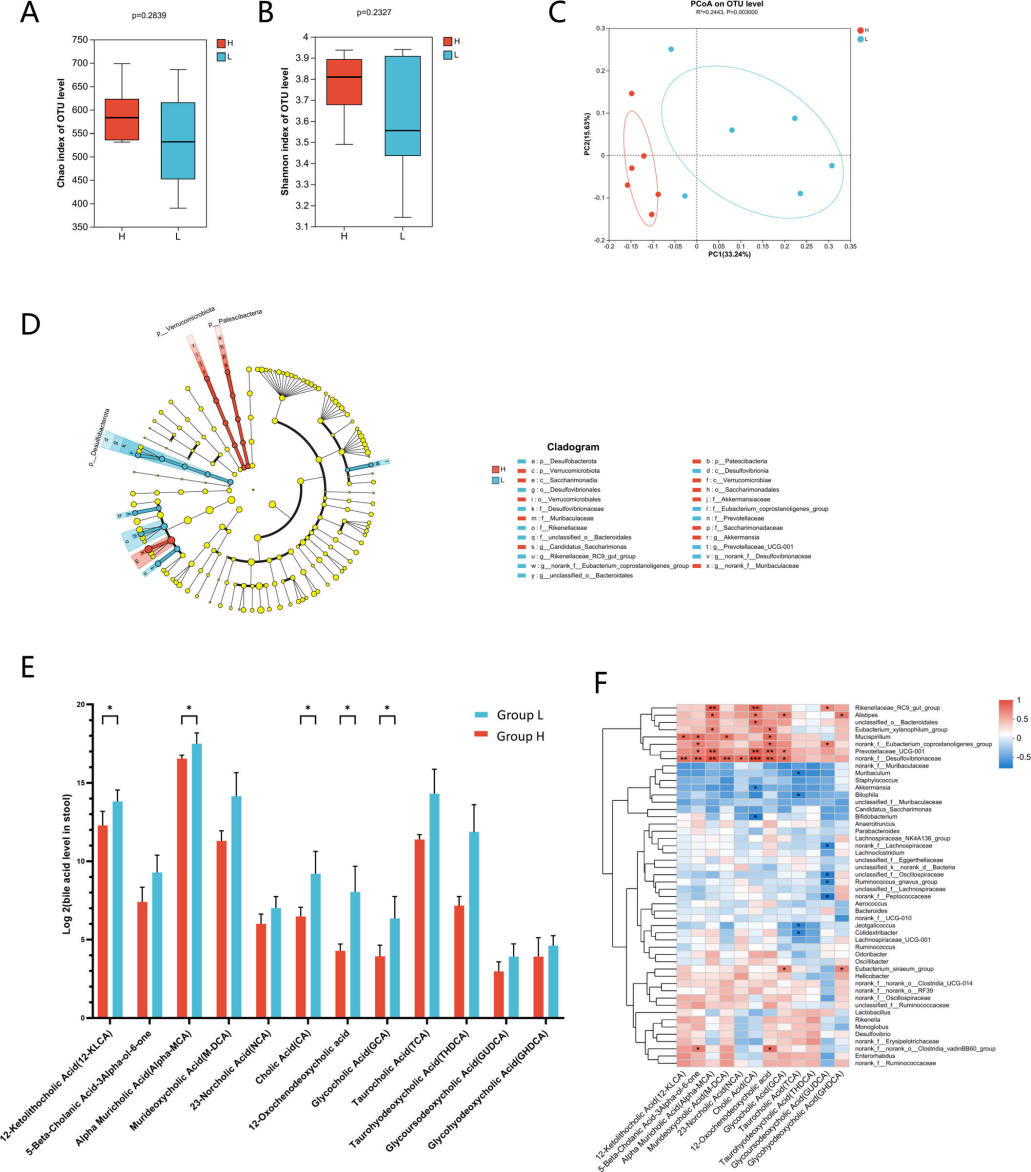
Gut microbiota and bile acid profile in CCl4-induced mice after stool gavage. Alpha diversity indexes (**A**) Chao1 and (**B**) Shannon. (**C**) PCoA is based on Bray-Curtis. (**D**) LEfSe cladogram represents the taxa enriched in the groups H and L. (**E**) Levels of fecal bile acids in CCl4-induced mice gavaged stool from patients with hepatitis B-related cirrhosis or stool from healthy controls. (**F**) The correlation analysis between fecal bile acid concentrations and gut microbial abundance (genus level). Group NC: negative controls, mice without CCl4 and fecal microbiota transplantation. Group L: fecal microbiota transplantation from HBV-related cirrhosis patients; Group H: FMT from age- and gender-matched healthy controls as the cirrhotic patients; Group PC: positive controls, only CCl4 was applied to induce fibrosis, and FMT was not performed.* p < 0.05; **P < 0.01.

To investigate the direct impact of cirrhotic gut microbiota on hepatic fibrosis, 16S rRNA sequencing was performed on fecal samples from FMT-treated mice. The Chao1 (Fig. 5A) and Shannon (Fig. 5B) indices showed no significant differences in the overall microbial diversity between Group H (FMT from healthy controls) and Group L (FMT from cirrhosis patients). However, Bray-Curtis-based PCoA revealed distinct clustering patterns, indicating significant differences in gut microbial composition between the two groups (Fig. 5C).

Linear discriminant analysis effect size identified key taxa differentiating the groups (Fig. 5D), with Group L showing enrichment of phylum *Desulfobacterota*; families *Rikenellaceae*, *Prevotellaceae*, and *Desulfovibrionaceae*; genus *Bacteroidales*, *Eubacterium*. Conversely, Group H exhibited higher abundances of phylum Verrucomicrobia, Patescibacteria; families *Muribaculaceae*, *Akkermansiaceae*, and *Saccharimonadaceae*; genus *Akkermansia*, *Candidatus*, and *Muribaculaceae*. These results indicate that FMT from HBV-related cirrhosis patients induces specific alterations in gut microbial composition, contributing to the progression of hepatic fibrosis.

### Disturbed bile acid metabolism and microbiota-bile acid crosstalk

Bile acid profiling revealed significantly higher fecal concentrations of Alpha-MCA, 12-ketolithocholic acids (12-KLCA), cholic acid (CA), glycochenodeoxycholic acid (GCA), and 12-oxochenodeoxycholic acid in Group L compared to Group H (Fig. 5E). To explore bile acid-microbiota interactions, correlation analyses were performed between fecal bile acid levels and microbial taxa (genus level). Notably, a positive correlation was observed between the relative abundance of *Desulfovibrionaceae* and elevated bile acid levels: 12-KLCA, 5-beta-cholanic acid-3alpha-ol-6-one, alpha-MCA, murideoxycholic Acid (M-DCA), norcholic acid (NCA), CA, 12-oxochenodeoxycholic acid, and GCA. On the other hand, a negative correlation existed between *Akkermansia* abundance and bile acid concentrations, especially CA. These findings suggest that FMT from HBV-related cirrhosis patients exacerbates hepatic fibrosis by promoting potential pathogen bacteria (*Desulfovibrionaceae*), reducing beneficial bacteria (*Akkermansia*), and disrupting bile acid metabolism (Fig. 5F).

## DISCUSSION

In this study, we established, for the first time, a correlation between liver pathology and gut microbiota in patients with CHB, identifying characteristic gut microbial signatures associated with hepatic inflammation and fibrosis. Furthermore, we demonstrated that FMT from patients with hepatitis B-related cirrhosis into a murine model of hepatic fibrosis exacerbated disease progression. This effect appears to be related to alterations in gut microbiota composition and bile acid metabolism. Our findings provide the first direct evidence that gut microbiota can drive hepatitis B-associated hepatic fibrosis.

Previous studies have shown that gut microbial composition varies according to HBV-induced serum ALT levels. For example, *Desulfovibrio* and *Megasphaera* showed positive correlations, while *Acidaminococcus* exhibited a negative correlation with elevated ALT levels in healthy HBV carriers (15). Another pilot study found that HBsAg-positive patients with high ALT levels had significantly lower counts of *Lactobacillus* compared to those with normal ALT levels (16). Consistent with the Korean cohort study (15), our analysis using ANOSIM revealed significantly lower intragroup diversity among patients with significant histological damage, suggesting a more homogeneous microbial profile in these individuals. However, we did not observe differences in the above-mentioned bacterial genera between the two groups of this study. This might be due to differences in detection methods. It is also possible that our queue patients did not have a significant increase in liver enzyme levels. Even in patients with significant histological damage (Group S), the liver enzyme levels were only slightly above the normal range (Table 2). Furthermore, these previous studies included patients with comorbidities such as fatty liver disease and diabetes, which could confound the association between HBV infection and liver damage. In contrast, our study focused on untreated individuals with isolated HBV infection and assessed liver inflammation and fibrosis through liver biopsy, excluding confounding factors like fatty liver disease. This approach enabled a more accurate reflection of the relationship between chronic HBV-induced inflammation and gut microbiota composition.

We observed reduced gut microbiota diversity in patients with significant liver pathology. Interestingly, patients with advanced liver injury exhibited an enrichment of *Bacteroides*, typically dominant residents of the gut microbiota. Previous studies have reported inconsistent findings regarding *Bacteroides* in HBV-related liver fibrosis. For example, reductions in *Bacteroides* were observed in CHB patients compared to healthy controls (17, 18). These discrepancies may be due to differences in comparison groups. While earlier studies compared CHB patients with healthy controls, we compared HBV carriers with varying degrees of liver injury. Notably, in HCV-infected patients, *Bacteroides vulgatus* showed increased transcriptional activity related to liver disease severity despite no significant difference in the relative abundance (19). Some *Bacteroides* species, such as *B. vulgatus*, *B. uniformis*, *and B. ovatus*, have been linked to negative effects on energy and immune homeostasis in nonalcoholic fatty liver disease (20) and hepatic disorder. Studies have shown that in patients with non-alcoholic fatty liver disease (NAFLD), the abundance of *Bacteroides* is significantly correlated with NASH-related liver injury. It has been hypothesized that *Bacteroides* may exacerbate the progression of NAFLD by regulating multiple metabolic pathways. Notably, our metagenomic sequencing results revealed that more severe liver tissue damage in CHB patients is associated with gut microbial metabolic functions, particularly those involving Carbohydrate Metabolism, Glycan Biosynthesis and Metabolism, Metabolism of Cofactors and Vitamins, and Transport and Catabolism. These compelling findings provide novel mechanistic insights into the potential role of gut microbiota in the pathogenesis of CHB. The identification of microbial-derived metabolic regulators opens new avenues for understanding disease chronicity. Further systematic validation is required to precisely determine which microbial metabolic pathways are directly related to CHB-associated histopathological changes. Compared to 16S rRNA-based sequencing approaches, the metagenomic methodology employed in this CHB study cohort demonstrates advantages in taxonomic resolution, enabling specific identification of functionally differentiated Bacteroides species. Nevertheless, it is crucial to emphasize that this approach carries inherent limitations, including financial burden and limited accessibility, time-consuming in analysis, and the potential introduction of host-derived contaminants (21, 22).

A key question is whether gut microbiota dysbiosis is merely a secondary consequence of liver inflammation or a driver of hepatic inflammation and fibrosis progression. Although symbiotic gut microbes provide numerous benefits, such as modulating the immune system and protecting against pathogens, dysbiosis can promote disease development (23, 24). Despite increasing evidence linking gut microbiota to liver fibrosis progression, the causal relationship in HBV-related fibrosis remains unclear. Some argue that hepatic inflammation triggers gut dysbiosis, while others suggest that dysbiosis contributes to liver disease progression. Population-based studies cannot fully resolve this issue. Animal models provide critical insights into causality. Previous studies in pseudo-germ-free mice demonstrated the pro-fibrotic effects of gut microbiota in bile duct ligation-induced liver fibrosis (25). FMT from patients with primary biliary cholangitis has also been shown to worsen liver injury in mice (26). In our study, FMT from HBV-cirrhosis patients led to increased fibroproliferation and extracellular matrix accumulation in mice, along with elevated expression of proinflammatory cytokines. These findings suggest that specific gut microbiota compositions can directly promote hepatic fibrosis in the context of chronic HBV infection.

We hypothesize that gut microbiota-bile acid crosstalk is a key mechanism driving HBV-related liver fibrosis. Bile acids are end products of cholesterol metabolism and play critical roles as signaling molecules regulated by the gut microbiota (27). Disruption of bile acid homeostasis has been implicated in chronic liver diseases. Abnormal bile acid levels are associated with hepatic fibrosis progression, as shown by studies demonstrating that unconjugated cholic acid promotes hepatic stellate cell activation (28, 29). Bile acids can directly alter the gut microbiota by disrupting bacterial membranes and modulating lipid structures (30). In our study, mice receiving FMT from cirrhotic patients exhibited elevated fecal BA levels, particularly unconjugated bile acids such as GCA and CA. These findings align with those of previous studies showing increased serum bile acid levels in patients with chronic hepatitis B-related liver failure (28). Additionally, correlation analyses revealed strong associations between bile acid levels and specific gut bacteria, such as *Parabacteroides distasonis*, which modulates fibrosis through bile acid metabolism (29, 31). Our results support the hypothesis that gut dysbiosis in CHB may exacerbate hepatic fibrosis via gut microbiota-bile acid interactions. Building on the emerging evidence of probiotics and their metabolites combined interventions enhancing HBsAg clearance (32), we propose targeted microbiome remodeling via multi-strain consortia transplantation and metabolic pathway modulation as a promising approach to mitigate CHB-related liver damage.

### Limitations

Our study has several limitations that warrant consideration. First, the relatively small patient cohort may have limited the identification of additional relevant microbial markers. Second, healthy controls were excluded based on the well-documented gut microbiota differences between CHB patients and healthy populations (10, 33–35). While this focused design facilitated precise exploration of microbial variations associated with liver injury progression, we explicitly acknowledge that subsequent large-scale validation studies will systematically incorporate healthy controls to strengthen comparative analyses through inter-group assessments. Third, while our animal experiments demonstrated that gut microbiota promotes hepatic fibrosis progression potentially through bile acid metabolism, we did not identify specific bacterial species or elucidate the precise molecular pathways involved. Future research should focus on identifying key microbial taxa and uncovering the mechanisms underlying gut microbiota-bile acid crosstalk in hepatic fibrosis. Fourth, given the virological characteristics of our cohort derived from a region with HBV genotype C predominance (co-circulating with other genotypes and intergenotypic recombinants), the generalizability of our findings requires cautious interpretation across distinct viral genotypes. Emerging evidence (36) suggests that host-pathogen interactions may vary substantially among HBV genotypes, potentially influencing disease progression trajectories and histopathological outcomes. This limitation will be addressed through multi-regional validation frameworks in follow-up studies.

### Conclusion

This study provides compelling evidence that gut microbiota dysbiosis contributes to hepatic fibrosis during chronic HBV infection, with bile acid metabolism playing a potential mediating role. By utilizing a humanized gut microbiota mouse model, we highlight the fibrogenic impact of microbial imbalance and bile acid metabolic disorders. These findings highlight the potential therapeutic value of modulating gut microbiota dysbiosis to mitigate the progression of CHB. Further research is needed to elucidate the complex interactions between key microbial species, host metabolism, and liver fibrosis progression.
